# Di-μ-nicotinato-κ^2^ *N*:*O*;κ^2^ *O*:*N*-bis­[aqua­(ethyl­enediamine-κ^2^ *N*,*N*′)(nicotinato-κ*N*)cadmium(II)] dihydrate

**DOI:** 10.1107/S1600536808009756

**Published:** 2008-04-16

**Authors:** Jan Moncol, Dušan Mikloš, Peter Segla, Marian Koman

**Affiliations:** a Department of Inorganic Chemistry, Slovak Technical University, Radlinského 9, SK-812 37 Bratislava, Slovakia

## Abstract

The dinuclear mol­ecule of the title compound, [Cd_2_(C_6_H_4_NO_2_)_4_(C_2_H_8_N_2_)_2_(H_2_O)_2_]·2H_2_O, lies on an inversion centre and forms 12-membered (CdNC_3_O)_2_ metallacycles with the two Cd^2+^ ions bridged by two nicotinate ligands. Both Cd^2+^ ions display coordination polyhedra with a distorted octa­hedral geometry that includes two pyridine N atoms from bridging and terminal nicotinate anions, two amine N atoms from chelating ethylene­diamine ligands, carboxylate O atoms from bridging nicotinate anions and water O atoms. Inter­molecular O—H⋯O and N—H⋯O hydrogen bonds result in the formation of a three-dimensional network, and π–π stacking inter­actions are observed between symmetry-related pyridine rings of bridging as well as terminal nicotinate anions (the centroid–centroid distances are 3.59 and 3.69 Å, respectively, and the distances between parallel planes of the stacked pyridine rings are 3.53 and 3.43 Å, respectively). The two methylene groups of the ethylene­diamine ligand are disordered over two positions; the site occupancy factors are *ca* 0.8 and 0.2.

## Related literature

For related literature, see: Bernstein *et al.* (1995 ▶); Chen (2003 ▶); Clegg *et al.* (1995 ▶); Evans & Lin (2001 ▶); Janiak (2000 ▶); Kang *et al.* (2007 ▶); Liang & Li (2005 ▶); Lu & Kohler (2002 ▶); Lu *et al.* (2007 ▶); Luo *et al.* (2004 ▶); Song *et al.* (2006 ▶); Xian *et al.* (2007 ▶); Zhang *et al.* (1996 ▶); Zhang *et al.* (2004 ▶). For related structures, see: Ayyappan *et al.* (2001 ▶); Abu-Youssef (2005 ▶); Chen *et al.* (2001 ▶, 2008 ▶); Lin *et al.* (2000 ▶); Liu *et al.* (2005 ▶); Madalan *et al.* (2005 ▶); Wang *et al.* (2002 ▶); Wasson & LaDuca (2007 ▶); Wu *et al.* (2003 ▶).
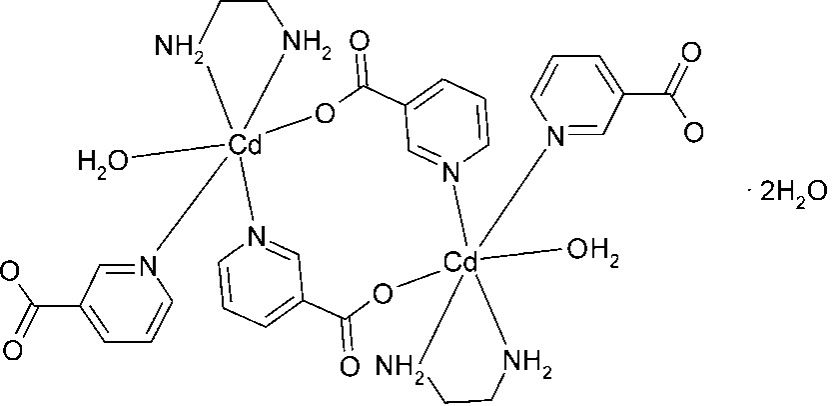


## Experimental

### 

#### Crystal data

[Cd_2_(C_6_H_4_NO_2_)_4_(C_2_H_8_N_2_)_2_(H_2_O)_2_]·2H_2_O
*M* *_r_* = 905.18
Triclinic, 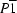

*a* = 7.678 (1) Å
*b* = 10.364 (1) Å
*c* = 11.984 (2) Å
α = 101.080 (1)°
β = 93.60 (1)°
γ = 109.63 (1)°
*V* = 873.1 (2) Å^3^
*Z* = 1
Mo *K*α radiation
μ = 1.29 mm^−1^
*T* = 294 (2) K
0.35 × 0.30 × 0.20 mm

#### Data collection

Siemens *P*4 diffractometer
Absorption correction: ψ scan (*XEMP*; Siemens, 1994 ▶) *T*_min_ = 0.652, *T*_max_ = 0.776
6118 measured reflections
5071 independent reflections
4491 reflections with *I* > 2σ(*I*)
*R*_int_ = 0.055
3 standard reflections every 97 reflections intensity decay: 2.0%

#### Refinement

*R*[*F* ^2^ > 2σ(*F* ^2^)] = 0.030
*wR*(*F* ^2^) = 0.076
*S* = 1.06
5071 reflections
245 parameters
21 restraints
H-atom parameters constrained
Δρ_max_ = 0.57 e Å^−3^
Δρ_min_ = −0.62 e Å^−3^

### 

Data collection: *XSCANS* (Siemens, 1994 ▶); cell refinement: *XSCANS*; data reduction: *XSCANS*; program(s) used to solve structure: *SHELXS97* (Sheldrick, 2008 ▶); program(s) used to refine structure: *SHELXL97* (Sheldrick, 2008 ▶); molecular graphics: *ORTEP-3* (Farrugia, 1997 ▶); software used to prepare material for publication: *enCIFer* (Allen *et al.*, 2004 ▶).

## Supplementary Material

Crystal structure: contains datablocks global, I. DOI: 10.1107/S1600536808009756/zl2107sup1.cif

Structure factors: contains datablocks I. DOI: 10.1107/S1600536808009756/zl2107Isup2.hkl

Additional supplementary materials:  crystallographic information; 3D view; checkCIF report

## Figures and Tables

**Table 1:** Hydrogen-bond geometry (Å, °)

*D*—H⋯*A*	*D*—H	H⋯*A*	*D*⋯*A*	*D*—H⋯*A*
O1*W*—H1*W*⋯O4^i^	0.82	1.84	2.659 (2)	174
O1*W*—H2*W*⋯O2^ii^	0.84	1.93	2.762 (3)	169
O2*W*—H3*W*⋯O2*W*^iii^	0.84	2.25	3.041 (10)	158
O2*W*—H4*W*⋯O3^iv^	0.82	1.97	2.742 (5)	157
N3—H3*A*⋯O2	0.89	2.37	3.099 (3)	139
N3—H3*B*⋯O4^v^	0.90	2.11	2.966 (3)	160
N3—H3*C*⋯O2	0.89	2.36	3.099 (3)	141
N3—H3*D*⋯O4^v^	0.90	2.24	2.966 (3)	138
N4—H4*A*⋯O3^i^	0.91	2.16	3.054 (3)	169
N4—H4*B*⋯O2*W*	0.91	2.22	2.975 (4)	140
N4—H4*C*⋯O3^i^	0.92	2.26	3.054 (3)	145
N4—H4*D*⋯O2*W*	0.90	2.13	2.975 (4)	157

Symmetry codes: (i) 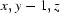; (ii) 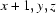; (iii) 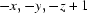; (iv) 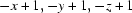; (v) 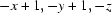.

**Fig. 1.**
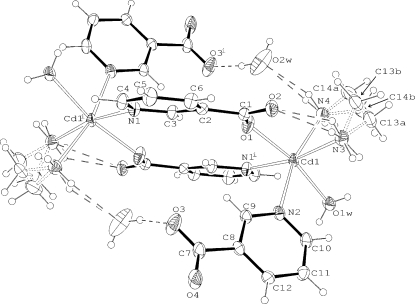
Perspective view of (I), with the atom numbering scheme. Thermal ellipsoids are drawn at the 30% probability level. Bonds in the minor disordered parts are drawn as open-dashed lines.

**Fig. 2.**
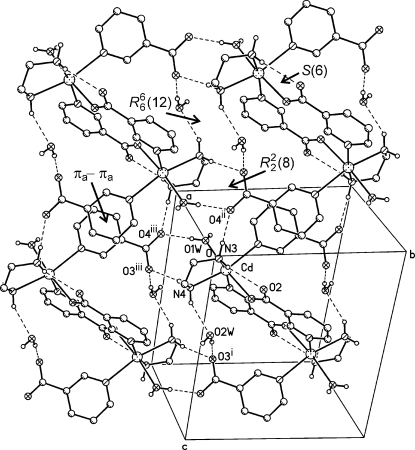
The hydrogen-bonds and πa-πa stacking interactions in the crystal packing of (I). Only the major disordered part is shown. [symmetry codes: (i) -x + 1, -y + 1, -z + 1; (ii) -x + 1, -y + 1, -z; (iii) x, y - 1, z]

**Fig. 3.**
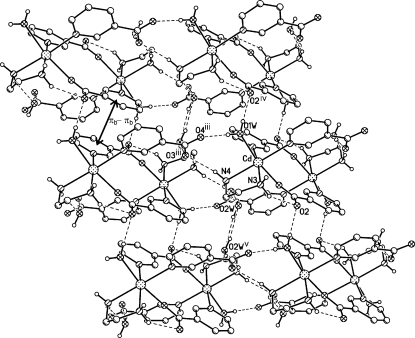
The hydrogen-bonds and πb-πb stacking interactions in the crystal packing of (I). Only the major disordered part is shown. [symmetry codes: (iii) x, y - 1, z; (iv) x + 1, y, z; (v) -x, -y, -z + 1]

## supplementary crystallographic information

### Comment

Several Cd^II^ coordination polymers that contain bridging 3-pyridylcarboxylate (nicotinate) ligands have been reported recently (Abu-Youssef, 2005; Evans & Lin, 2001; Chen, (2003); Clegg *et al.*, 1995; Kang *et al.*, 2007; Liang & Li, 2005; Lu & Kohler, 2002; Lu *et al.*, 2007; Song *et al.*, 2006; Xian *et al.* 2007; Zhang *et al.*, 1996; Zhang *et al.*, 2004). However, if the nicotinate anions are coordinated only as terminal ligands, the possibility of participating in a hydrogen-bonding network originates. As part of our efforts to investigate metal(II) complexes based on pyridine carboxylic acids, we report herein the crystal structure of the title compound, (I).

Figure 1 shows a representative *ORTEP* diagram for (I). The Cd centers are µ_2_-bridged by two nicotinate ligands to form a twelve-membered (CdNC_3_O)_2_ ring with the Cd···Cd^i^ [symmetry code: (i) -*x* + 1, -*y* + 1, -*z* + 1] distance being 7.355 (1) Å. The two nicotinate ligands bridge two Cd centers through the pyridyl N atom and one of the carboxylate O atoms. The Cd^2+^ ion has a distorted octahedral coordination formed by the two pyridine N atoms of bridging [Cd–N1^i^ = 2.349 (2) Å] and terminal [Cd–N2 = 2.406 (2) Å] nicotinate anions; the two N atoms of chelating 1,2-ethylenediamine ligands [Cd–N3 = 2.321 (2) Å and Cd–N4 = 2.345 (2) Å], and two O atoms in *trans* positions, one from the carboxylate group of a µ_2_-bridging nicotinate ligand [Cd–O1 = 2.325 (2) Å] and one from the coordinated water molecule [Cd–O1W = 2.348 (2) Å].

The structure of (I) can be compared with two dimeric copper(II) complexes with a µ_2_-bridging nicotinate ligand [Cu(µ_2_-nic)(dien)]_2_(nic)_2_ (II) and [Cu(µ_2_-nic)(dien)]_2_(BF_4_)_2_.2MeOH (III) [dien is diethylenetriamine] (Chen *et al.*, 2008). Both compounds (II-III) are dimeric complexes, where the nicotinate ligands are bridging two Cu centers to form similar twelve-membered (CuNC_3_O)_2_ rings (Table 2). On the other hand, the same (MNC_3_O)_2_ [M = Cu, Cd, Ni or Mn] rings are observed in some metal(II) nicotinate based coordination polymers, but the nicotinate ligands are µ_3_-bridging ones. M···M distances and chromophores for dinuclear and polymeric complexes with twelve-membered (MNC_3_O)_2_ rings are compared in Table 2.

The hydrogen-bonding parameters of (I) are listed in Table 1. In the crystal structure, intermolecular O–H···O and N–H···O hydrogen-bonds (Table 1) link the molecules to form a three-dimensional network (Figures 2 and 3). One of the amine H atoms of the 1,2-ethylenediamine ligand forms an intramolecular hydrogen-bond [N3–H3A···O2 or N3–H3C···O2] and partipicates in creation of aan intramolecular metallocyclus with an *S*(6) pattern (Bernstein *et al.*, 1995) (Figure 2). One O atom and one H atom of each of the uncoordinated water molecules, two amine groups of the 1,2-ethylenediamine ligands and one carboxylate O atom of each of the terminal nicotinate ligands are connected through hydrogen-bonds to rings with a graph set motif of *R*_6_^6^(12) (Bernstein *et al.*, 1995) [N4–H4B···O2W or N4–H4D···O2W; N4–H4A···O3^iii^ or N4–H4C···O3^iii^; and O2W–H4W···O3^i^, symmetry codes: (i) -*x* + 1, -*y* + 1, -*z* + 1; (iii) *x*, *y* - 1, *z*] (Figure 2). The H atoms from the amine groups of the 1,2-ethylenediamine ligand and the H atoms from the coordinated water molecule are connected through hydrogen-bonds to the carboxylate O atoms of the terminal nicotinate ligands [N4–H4A···O3^iii^ or N4–H4C···O3^iii^; O1W–H1W···O4^iii^] and these groups create six-membered *R*_2_^2^(8) rings (Bernstein *et al.*, 1995) (Figure 2). The remaining hydrogen-bonds from the amine groups of the ethylendiamine ligands are connected to carboxylate O atoms of terminal nicotinate ligands of neighbouring complex molecules [N3–H3B···O4^ii^ or N3–H3D···O4^ii^, symmetry codes: (ii) -*x* + 1, -*y* + 1, -*z*] (Figure 2). Further intermolecular hydrogen-bonds between uncoordinated water molecules [O2W–H3W···O2W^v^, symmetry codes: (v) -*x*, -*y*, -*z* + 1], and between coordinated water molecules and carboxylate O atoms of bridging nicotinate ligands [O1W–H2W···O2^iv^, symmetry codes: (iv) *x* + 1, *y*, *z*] are shown in Figure 3.

Additional interactions between the pyridine rigs of nicotinate ligands are π-π stacking interactions (Janiak, 2000) between the two adjacent pyridine rings of terminal nicotinate ligands [N2/C8—C12] (π_a_-π_a_^ii^) (Figure 2), and between the two adjacent pyridine rings of bridging nicotinate ligands [N1/C2—C6] (π_b_-π_b_^vi^) (Figure 3) [symmetry codes: (ii) -*x* + 1, -*y* + 1, -*z*; (vi) -*x*, -*y* + 1, -*z* - 1]. The centroid (C_g_) distances C_g_···C_g_ are 3.69 and 3.59 Å, respectively. The distances between parallel planes of the stacked pyridine rings are 3.43 and 3.53 Å, respectively.

### Experimental

The title complex was formed in a methanolic solution (30 cm^3^) of Cd(nicotinate)_2_.H_2_O (1.25 mmol) by adding 1,2-ethylenediamine in the molar ratio of 1:1. The resulting solution was left to slowly evaporate at room temperature. Well shaped crystals, suitable for X-ray structure analysis were collected after a few days by filtration and finally dried *in vacuo*. Anal. Calc.: C, 37.14; H, 4.45; N, 12.37; Cd, 24.83; Found: C, 36.82; H, 4.53; N, 12.30; Cd, 24.65. Selected IR data (KBr) cm^-1^: 1611 *vs*,br ν_as_(COO^-^); 1383 *vs*,br ν_s_(COO^-^); 643*m*δ(pyridine ring in-plane bending); 432*m*χ(pyridine ring out-of-plane bending).

### Refinement

The 1,2-ethylenediamine ligand has orientational disorder [C13A—C14A and C13B—C14B] and the refined site-occupancy factors of both the disordered parts are 0.78 (1) and 0.22 (1), respectively. The disordered parts of the title compounds were restrained using SADI, DELU and SIMU commands (*SHELXL97*; Sheldrick, 2008). All H atoms of C–H (aromatic, methylene) and N–H (amine) bonds were placed in calculated positions (0.93, 0.97 and 0.89–0.92 Å, respectively); isotropic displaced parameters were fixed (*U*_iso_(H) = 1.2 *U*_iso_(C/N) of C or N atoms to which they were attached) using a riding model. The water H atoms were placed in calculed positions (O–H = 0.82–0.84 Å); isotropic displaced parameters were fixed (*U*_iso_(H) = 1.5 *U*_iso_(O)) of O atoms to which they were attached).

### Crystal data

**Table:**

[Cd_2_(C_6_H_4_NO_2_)_4_(C_2_H_8_N_2_)_2_(H_2_O)_2_]·2H_2_O	*Z* = 1
*M**_r_* = 905.18	*F*_000_ = 456
Triclinic, *P*1	*D*_x_ = 1.722 Mg m^−^^3^
Hall symbol: -P1	Mo *K*α radiation λ = 0.71073 Å
*a* = 7.6780 (10) Å	Cell parameters from 25 reflections
*b* = 10.3640 (10) Å	θ = 2.1–8.9º
*c* = 11.984 (2) Å	µ = 1.29 mm^−^^1^
α = 101.080 (10)º	*T* = 294 (2) K
β = 93.600 (10)º	Prism, colourless
γ = 109.630 (10)º	0.35 × 0.30 × 0.20 mm
*V* = 873.1 (2) Å^3^	

### Data collection

**Table:**

Siemens P4 diffractometer	*R*_int_ = 0.055
Radiation source: fine-focus sealed tube	θ_max_ = 30.0º
Monochromator: graphite	θ_min_ = 1.8º
*T* = 294(2) K	*h* = −1→10
2θ/ω scans	*k* = −14→13
Absorption correction: ψ scan(XEMP; Siemens, 1994)	*l* = −16→16
*T*_min_ = 0.652, *T*_max_ = 0.776	3 standard reflections
6118 measured reflections	every 97 reflections
5071 independent reflections	intensity decay: 2.0%
4491 reflections with *I* > 2σ(*I*)	

### Refinement

**Table:**

Refinement on *F*^2^	Secondary atom site location: difference Fourier map
Least-squares matrix: full	Hydrogen site location: inferred from neighbouring sites
*R*[*F*^2^ > 2σ(*F*^2^)] = 0.030	H-atom parameters constrained
*wR*(*F*^2^) = 0.076	*w* = 1/[σ^2^(*F*_o_^2^) + (0.0307*P*)^2^ + 0.1604*P*] where *P* = (*F*_o_^2^ + 2*F*_c_^2^)/3
*S* = 1.06	(Δ/σ)_max_ = 0.001
5071 reflections	Δρ_max_ = 0.57 e Å^−^^3^
245 parameters	Δρ_min_ = −0.62 e Å^−^^3^
21 restraints	Extinction correction: none
Primary atom site location: structure-invariant direct methods	

### Special details

**Table:**

Geometry. All esds (except the esd in the dihedral angle between two l.s. planes) are estimated using the full covariance matrix. The cell esds are taken into account individually in the estimation of esds in distances, angles and torsion angles; correlations between esds in cell parameters are only used when they are defined by crystal symmetry. An approximate (isotropic) treatment of cell esds is used for estimating esds involving l.s. planes.
Refinement. Refinement of F^2^ against ALL reflections. The weighted R-factor wR and goodness of fit S are based on F^2^, conventional R-factors R are based on F, with F set to zero for negative F^2^. The threshold expression of F^2^ > 2sigma(F^2^) is used only for calculating R-factors(gt) etc. and is not relevant to the choice of reflections for refinement. R-factors based on F^2^ are statistically about twice as large as those based on F, and R- factors based on ALL data will be even larger.

### Fractional atomic coordinates and isotropic or equivalent isotropic displacement parameters (Å^2^)

**Table:**

	*x*	*y*	*z*	*U*_iso_*/*U*_eq_	Occ. (<1)
Cd1	0.50747 (2)	0.217937 (14)	0.263617 (13)	0.03264 (6)	
O1	0.3261 (3)	0.3353 (2)	0.35364 (17)	0.0505 (5)	
O1W	0.7358 (3)	0.13999 (17)	0.18352 (18)	0.0457 (4)	
H1W	0.7166	0.0558	0.1631	0.069*	
H2W	0.8505	0.1895	0.2005	0.069*	
O2	0.1020 (3)	0.3222 (2)	0.21860 (16)	0.0471 (4)	
O2W	0.1968 (6)	0.0994 (5)	0.4981 (3)	0.1256 (15)	
H3W	0.1050	0.0314	0.5055	0.188*	
H4W	0.2579	0.1496	0.5595	0.188*	
O3	0.6053 (4)	0.8065 (2)	0.2856 (2)	0.0739 (8)	
O4	0.6980 (4)	0.87012 (19)	0.12727 (19)	0.0619 (6)	
N1	0.2917 (3)	0.6858 (2)	0.56161 (17)	0.0364 (4)	
N2	0.6293 (3)	0.41008 (18)	0.17134 (17)	0.0353 (4)	
N3	0.2678 (3)	0.0997 (2)	0.11238 (19)	0.0429 (4)	
H3A	0.1697	0.1239	0.1265	0.051*	0.780 (10)
H3B	0.3071	0.1225	0.0476	0.051*	0.780 (10)
H3C	0.2002	0.1532	0.1062	0.051*	0.220 (10)
H3D	0.3192	0.0863	0.0479	0.051*	0.220 (10)
N4	0.3717 (3)	−0.0048 (2)	0.30277 (19)	0.0445 (5)	
H4A	0.4522	−0.0522	0.2930	0.053*	0.780 (10)
H4B	0.3529	0.0051	0.3776	0.053*	0.780 (10)
H4C	0.4641	−0.0346	0.3276	0.053*	0.220 (10)
H4D	0.3025	0.0006	0.3600	0.053*	0.220 (10)
C1	0.2052 (3)	0.3777 (2)	0.3123 (2)	0.0347 (4)	
C2	0.1904 (3)	0.5085 (2)	0.38534 (19)	0.0315 (4)	
C3	0.2937 (3)	0.5680 (2)	0.4933 (2)	0.0357 (4)	
H3	0.3691	0.5233	0.5199	0.043*	
C4	0.1810 (4)	0.7494 (2)	0.5236 (2)	0.0412 (5)	
H4	0.1777	0.8311	0.5702	0.049*	
C5	0.0722 (4)	0.6972 (3)	0.4178 (2)	0.0468 (6)	
H5	−0.0040	0.7429	0.3940	0.056*	
C6	0.0771 (3)	0.5761 (3)	0.3470 (2)	0.0407 (5)	
H6	0.0058	0.5405	0.2749	0.049*	
C7	0.6597 (4)	0.7856 (2)	0.1908 (2)	0.0428 (5)	
C8	0.6792 (3)	0.6441 (2)	0.1471 (2)	0.0326 (4)	
C9	0.6207 (3)	0.5384 (2)	0.20765 (19)	0.0333 (4)	
H9	0.5737	0.5576	0.2761	0.040*	
C10	0.6977 (3)	0.3842 (2)	0.0733 (2)	0.0386 (5)	
H10	0.7041	0.2955	0.0478	0.046*	
C11	0.7595 (4)	0.4825 (3)	0.0076 (2)	0.0399 (5)	
H11	0.8065	0.4605	−0.0603	0.048*	
C12	0.7494 (3)	0.6147 (2)	0.0455 (2)	0.0358 (4)	
H12	0.7895	0.6829	0.0030	0.043*	
C13A	0.2200 (8)	−0.0529 (4)	0.1059 (4)	0.0548 (13)	0.780 (10)
H13A	0.3186	−0.0828	0.0767	0.066*	0.780 (10)
H13B	0.1052	−0.1059	0.0539	0.066*	0.780 (10)
C14A	0.1963 (8)	−0.0807 (5)	0.2237 (4)	0.0604 (14)	0.780 (10)
H14A	0.0978	−0.0504	0.2527	0.072*	0.780 (10)
H14B	0.1602	−0.1808	0.2193	0.072*	0.780 (10)
C13B	0.1473 (18)	−0.0289 (13)	0.1436 (17)	0.056 (4)	0.220 (10)
H13C	0.0625	−0.0923	0.0769	0.067*	0.220 (10)
H13D	0.0740	−0.0052	0.2019	0.067*	0.220 (10)
C14B	0.270 (2)	−0.0967 (12)	0.1881 (13)	0.052 (4)	0.220 (10)
H14C	0.3580	−0.1059	0.1354	0.062*	0.220 (10)
H14D	0.1959	−0.1898	0.1967	0.062*	0.220 (10)

### Atomic displacement parameters (Å^2^)

**Table:**

	*U*^11^	*U*^22^	*U*^33^	*U*^12^	*U*^13^	*U*^23^
Cd1	0.04196 (9)	0.02134 (7)	0.03364 (8)	0.01255 (6)	0.00412 (6)	0.00197 (5)
O1	0.0542 (11)	0.0569 (11)	0.0438 (9)	0.0363 (9)	−0.0027 (8)	−0.0076 (8)
O1W	0.0458 (9)	0.0247 (7)	0.0671 (12)	0.0154 (7)	0.0149 (8)	0.0040 (7)
O2	0.0444 (9)	0.0463 (9)	0.0415 (9)	0.0158 (8)	−0.0034 (7)	−0.0076 (7)
O2W	0.162 (4)	0.189 (4)	0.075 (2)	0.125 (3)	0.040 (2)	0.022 (2)
O3	0.135 (2)	0.0523 (12)	0.0625 (14)	0.0592 (14)	0.0451 (15)	0.0202 (11)
O4	0.1147 (19)	0.0276 (8)	0.0537 (12)	0.0330 (10)	0.0270 (12)	0.0143 (8)
N1	0.0429 (10)	0.0330 (9)	0.0338 (9)	0.0176 (8)	0.0026 (7)	0.0023 (7)
N2	0.0440 (10)	0.0227 (7)	0.0409 (10)	0.0147 (7)	0.0038 (8)	0.0067 (7)
N3	0.0484 (11)	0.0411 (10)	0.0368 (10)	0.0151 (9)	0.0021 (8)	0.0062 (8)
N4	0.0578 (13)	0.0339 (9)	0.0417 (11)	0.0138 (9)	0.0093 (9)	0.0124 (8)
C1	0.0322 (10)	0.0333 (10)	0.0352 (10)	0.0108 (8)	0.0063 (8)	0.0012 (8)
C2	0.0308 (9)	0.0300 (9)	0.0341 (10)	0.0113 (8)	0.0082 (8)	0.0059 (8)
C3	0.0391 (11)	0.0335 (10)	0.0361 (10)	0.0182 (9)	0.0038 (8)	0.0026 (8)
C4	0.0532 (14)	0.0342 (11)	0.0413 (12)	0.0234 (10)	0.0066 (10)	0.0060 (9)
C5	0.0578 (15)	0.0456 (13)	0.0460 (13)	0.0309 (12)	0.0013 (11)	0.0102 (11)
C6	0.0422 (12)	0.0442 (12)	0.0375 (11)	0.0198 (10)	0.0008 (9)	0.0068 (9)
C7	0.0629 (15)	0.0264 (9)	0.0439 (12)	0.0223 (10)	0.0083 (11)	0.0071 (9)
C8	0.0362 (10)	0.0228 (8)	0.0394 (10)	0.0122 (7)	0.0018 (8)	0.0064 (7)
C9	0.0402 (11)	0.0248 (9)	0.0367 (10)	0.0141 (8)	0.0050 (8)	0.0066 (8)
C10	0.0504 (13)	0.0262 (9)	0.0410 (11)	0.0191 (9)	0.0041 (9)	0.0028 (8)
C11	0.0485 (13)	0.0355 (11)	0.0404 (11)	0.0207 (10)	0.0128 (9)	0.0065 (9)
C12	0.0399 (11)	0.0279 (9)	0.0406 (11)	0.0112 (8)	0.0087 (9)	0.0102 (8)
C13A	0.069 (3)	0.0353 (17)	0.0425 (19)	0.0009 (17)	−0.0001 (18)	0.0024 (14)
C14A	0.062 (3)	0.048 (2)	0.052 (2)	−0.007 (2)	0.006 (2)	0.0158 (19)
C13B	0.043 (7)	0.043 (7)	0.063 (10)	0.004 (5)	−0.002 (6)	−0.004 (6)
C14B	0.055 (8)	0.024 (5)	0.064 (9)	0.001 (5)	0.011 (6)	0.003 (5)

### Geometric parameters (Å, °)

**Table:**

Cd1—N3	2.321 (2)	N4—H4D	0.8980
Cd1—O1	2.325 (2)	C1—C2	1.509 (3)
Cd1—N4	2.344 (2)	C2—C3	1.385 (3)
Cd1—O1W	2.348 (2)	C2—C6	1.394 (3)
Cd1—N1^i^	2.349 (2)	C3—H3	0.9300
Cd1—N2	2.406 (2)	C4—C5	1.378 (4)
O1—C1	1.264 (3)	C4—H4	0.9300
O1W—H1W	0.8187	C5—C6	1.387 (3)
O1W—H2W	0.8450	C5—H5	0.9300
O2—C1	1.247 (3)	C6—H6	0.9300
O2W—H3W	0.8365	C7—C8	1.519 (3)
O2W—H4W	0.8232	C8—C12	1.385 (3)
O3—C7	1.240 (3)	C8—C9	1.395 (3)
O4—C7	1.243 (3)	C9—H9	0.9300
N1—C3	1.339 (3)	C10—C11	1.384 (3)
N1—C4	1.344 (3)	C10—H10	0.9300
N1—Cd1^i^	2.349 (2)	C11—C12	1.388 (3)
N2—C10	1.334 (3)	C11—H11	0.9300
N2—C9	1.343 (3)	C12—H12	0.9300
N3—C13B	1.477 (12)	C13A—C14A	1.504 (6)
N3—C13A	1.484 (4)	C13A—H13A	0.9700
N3—H3A	0.8860	C13A—H13B	0.9700
N3—H3B	0.8955	C14A—H14A	0.9700
N3—H3C	0.8870	C14A—H14B	0.9700
N3—H3D	0.8982	C13B—C14B	1.479 (15)
N4—C14A	1.474 (5)	C13B—H13C	0.9700
N4—C14B	1.507 (12)	C13B—H13D	0.9700
N4—H4A	0.9105	C14B—H14C	0.9700
N4—H4B	0.9088	C14B—H14D	0.9700
N4—H4C	0.9180		
			
N3—Cd1—O1	90.64 (7)	O2—C1—C2	118.9 (2)
N3—Cd1—N4	76.38 (8)	O1—C1—C2	115.1 (2)
O1—Cd1—N4	100.83 (9)	C3—C2—C6	117.3 (2)
N3—Cd1—O1W	97.31 (8)	C3—C2—C1	120.63 (19)
O1—Cd1—O1W	169.36 (7)	C6—C2—C1	122.1 (2)
N4—Cd1—O1W	87.95 (7)	N1—C3—C2	124.0 (2)
N3—Cd1—N1^i^	168.76 (7)	N1—C3—H3	118.0
O1—Cd1—N1^i^	84.28 (7)	C2—C3—H3	118.0
N4—Cd1—N1^i^	94.70 (7)	N1—C4—C5	122.1 (2)
O1W—Cd1—N1^i^	89.07 (7)	N1—C4—H4	119.0
N3—Cd1—N2	91.32 (7)	C5—C4—H4	119.0
O1—Cd1—N2	88.34 (7)	C4—C5—C6	119.5 (2)
N4—Cd1—N2	164.61 (7)	C4—C5—H5	120.2
O1W—Cd1—N2	84.44 (7)	C6—C5—H5	120.2
N1^i^—Cd1—N2	98.52 (7)	C5—C6—C2	119.1 (2)
C1—O1—Cd1	130.80 (16)	C5—C6—H6	120.4
Cd1—O1W—H1W	120.5	C2—C6—H6	120.4
Cd1—O1W—H2W	121.2	O3—C7—O4	125.6 (2)
H1W—O1W—H2W	113.0	O3—C7—C8	117.7 (2)
H3W—O2W—H4W	113.8	O4—C7—C8	116.7 (2)
C3—N1—C4	118.0 (2)	C12—C8—C9	118.18 (19)
C3—N1—Cd1^i^	119.65 (15)	C12—C8—C7	121.6 (2)
C4—N1—Cd1^i^	122.28 (16)	C9—C8—C7	120.2 (2)
C10—N2—C9	117.98 (19)	N2—C9—C8	122.8 (2)
C10—N2—Cd1	117.60 (14)	N2—C9—H9	118.6
C9—N2—Cd1	124.23 (16)	C8—C9—H9	118.6
C13B—N3—Cd1	107.2 (6)	N2—C10—C11	123.4 (2)
C13A—N3—Cd1	107.0 (2)	N2—C10—H10	118.3
C13B—N3—H3A	80.3	C11—C10—H10	118.3
C13A—N3—H3A	109.8	C10—C11—C12	118.3 (2)
Cd1—N3—H3A	109.2	C10—C11—H11	120.8
C13B—N3—H3B	135.6	C12—C11—H11	120.8
C13A—N3—H3B	111.4	C8—C12—C11	119.4 (2)
Cd1—N3—H3B	109.0	C8—C12—H12	120.3
H3A—N3—H3B	110.3	C11—C12—H12	120.3
C13B—N3—H3C	107.7	N3—C13A—C14A	109.3 (4)
C13A—N3—H3C	133.5	N3—C13A—H13A	109.8
Cd1—N3—H3C	108.4	C14A—C13A—H13A	109.8
C13B—N3—H3D	115.9	N3—C13A—H13B	109.8
C13A—N3—H3D	86.8	C14A—C13A—H13B	109.8
Cd1—N3—H3D	108.1	H13A—C13A—H13B	108.3
H3C—N3—H3D	109.4	N4—C14A—C13A	110.5 (4)
C14A—N4—Cd1	108.3 (2)	N4—C14A—H14A	109.6
C14B—N4—Cd1	103.7 (6)	C13A—C14A—H14A	109.6
C14A—N4—H4A	110.7	N4—C14A—H14B	109.6
C14B—N4—H4A	85.4	C13A—C14A—H14B	109.6
Cd1—N4—H4A	109.4	H14A—C14A—H14B	108.1
C14A—N4—H4B	112.2	N3—C13B—C14B	107.7 (12)
C14B—N4—H4B	137.7	N3—C13B—H13C	110.2
Cd1—N4—H4B	109.4	C14B—C13B—H13C	110.2
H4A—N4—H4B	106.8	N3—C13B—H13D	110.2
C14A—N4—H4C	131.2	C14B—C13B—H13D	110.2
C14B—N4—H4C	110.1	H13C—C13B—H13D	108.5
Cd1—N4—H4C	109.3	C13B—C14B—N4	107.3 (12)
C14A—N4—H4D	88.0	C13B—C14B—H14C	110.3
C14B—N4—H4D	116.6	N4—C14B—H14C	110.3
Cd1—N4—H4D	109.8	C13B—C14B—H14D	110.3
H4C—N4—H4D	107.1	N4—C14B—H14D	110.3
O2—C1—O1	126.0 (2)	H14C—C14B—H14D	108.5
			
N3—Cd1—O1—C1	−33.5 (2)	O1—C1—C2—C3	−4.8 (3)
N4—Cd1—O1—C1	−109.7 (2)	O2—C1—C2—C6	−5.8 (3)
O1W—Cd1—O1—C1	105.0 (4)	O1—C1—C2—C6	173.7 (2)
N1^i^—Cd1—O1—C1	156.5 (2)	C4—N1—C3—C2	0.8 (4)
N2—Cd1—O1—C1	57.8 (2)	Cd1^i^—N1—C3—C2	−175.37 (17)
N3—Cd1—N2—C10	−65.68 (18)	C6—C2—C3—N1	−0.4 (4)
O1—Cd1—N2—C10	−156.28 (18)	C1—C2—C3—N1	178.1 (2)
N4—Cd1—N2—C10	−29.2 (4)	C3—N1—C4—C5	−0.2 (4)
O1W—Cd1—N2—C10	31.54 (18)	Cd1^i^—N1—C4—C5	175.8 (2)
N1^i^—Cd1—N2—C10	119.76 (17)	N1—C4—C5—C6	−0.6 (4)
N3—Cd1—N2—C9	109.32 (19)	C4—C5—C6—C2	0.9 (4)
O1—Cd1—N2—C9	18.72 (18)	C3—C2—C6—C5	−0.4 (4)
N4—Cd1—N2—C9	145.8 (3)	C1—C2—C6—C5	−179.0 (2)
O1W—Cd1—N2—C9	−153.46 (19)	O3—C7—C8—C12	−176.1 (3)
N1^i^—Cd1—N2—C9	−65.24 (19)	O4—C7—C8—C12	4.7 (4)
O1—Cd1—N3—C13B	−87.9 (8)	O3—C7—C8—C9	5.8 (4)
N4—Cd1—N3—C13B	13.1 (8)	O4—C7—C8—C9	−173.4 (3)
O1W—Cd1—N3—C13B	99.2 (8)	C10—N2—C9—C8	0.3 (3)
N1^i^—Cd1—N3—C13B	−25.0 (9)	Cd1—N2—C9—C8	−174.71 (16)
N2—Cd1—N3—C13B	−176.2 (8)	C12—C8—C9—N2	−0.2 (3)
O1—Cd1—N3—C13A	−121.2 (3)	C7—C8—C9—N2	178.0 (2)
N4—Cd1—N3—C13A	−20.2 (3)	C9—N2—C10—C11	−0.1 (4)
O1W—Cd1—N3—C13A	65.9 (3)	Cd1—N2—C10—C11	175.22 (19)
N1^i^—Cd1—N3—C13A	−58.3 (5)	N2—C10—C11—C12	−0.1 (4)
N2—Cd1—N3—C13A	150.4 (3)	C9—C8—C12—C11	−0.1 (3)
N3—Cd1—N4—C14A	−10.2 (3)	C7—C8—C12—C11	−178.2 (2)
O1—Cd1—N4—C14A	77.8 (3)	C10—C11—C12—C8	0.2 (4)
O1W—Cd1—N4—C14A	−108.2 (3)	C13B—N3—C13A—C14A	−46.9 (11)
N1^i^—Cd1—N4—C14A	162.9 (3)	Cd1—N3—C13A—C14A	48.5 (6)
N2—Cd1—N4—C14A	−47.9 (5)	C14B—N4—C14A—C13A	−46.1 (10)
N3—Cd1—N4—C14B	20.6 (7)	Cd1—N4—C14A—C13A	39.8 (6)
O1—Cd1—N4—C14B	108.7 (7)	N3—C13A—C14A—N4	−61.5 (8)
O1W—Cd1—N4—C14B	−77.4 (7)	C13A—N3—C13B—C14B	47.9 (10)
N1^i^—Cd1—N4—C14B	−166.3 (7)	Cd1—N3—C13B—C14B	−46.7 (16)
N2—Cd1—N4—C14B	−17.1 (8)	N3—C13B—C14B—N4	70 (2)
Cd1—O1—C1—O2	30.5 (4)	C14A—N4—C14B—C13B	49.7 (10)
Cd1—O1—C1—C2	−148.90 (17)	Cd1—N4—C14B—C13B	−53.2 (15)
O2—C1—C2—C3	175.7 (2)		

Symmetry codes: (i) −*x*+1, −*y*+1, −*z*+1.

### Hydrogen-bond geometry (Å, °)

**Table:**

*D*—H···*A*	*D*—H	H···*A*	*D*···*A*	*D*—H···*A*
O1W—H1W···O4^ii^	0.82	1.84	2.659 (2)	174
O1W—H2W···O2^iii^	0.84	1.93	2.762 (3)	169
O2W—H3W···O2W^iv^	0.84	2.25	3.041 (10)	158
O2W—H4W···O3^i^	0.82	1.97	2.742 (5)	157
N3—H3A···O2	0.89	2.37	3.099 (3)	139
N3—H3B···O4^v^	0.90	2.11	2.966 (3)	160
N3—H3C···O2	0.89	2.36	3.099 (3)	141
N3—H3D···O4^v^	0.90	2.24	2.966 (3)	138
N4—H4A···O3^ii^	0.91	2.16	3.054 (3)	169
N4—H4B···O2W	0.91	2.22	2.975 (4)	140
N4—H4C···O3^ii^	0.92	2.26	3.054 (3)	145
N4—H4D···O2W	0.90	2.13	2.975 (4)	157

Symmetry codes: (ii) *x*, *y*−1, *z*; (iii) *x*+1, *y*, *z*; (iv) −*x*, −*y*, −*z*+1; (i) −*x*+1, −*y*+1, −*z*+1; (v) −*x*+1, −*y*+1, −*z*.

### Table 2 Table 2. Comparative M···M distances (Å) and chromophores for selected dinuclear and polymeric complexes with 12-membered (MNC_3_O)_2_ rings

**Table:**

Compound	Bonding mode	M···M	Chromophore
I^a^	µ_2_-nic-κ^2^N:O	7.355 (1)	CdN_4_O_2_
II^b^	µ_2_-nic-κ^2^N:O	6.904 (2)	CuN_4_O
III^c^	µ_2_-nic-κ^2^N:O	6.972 (2)	CuN_4_O
IV^d^	µ_3_-nic-κ^3^N:O:O'	7.208 (1)	MnN_2_O_4_
V^e^	µ_3_-nic-κ^3^N:O:O'	7.304 (3)	CdN_2_O_4_
VI^f^	µ_3_-nic-κ^3^N:O:O'	6.736 (1)	CuN_3_O_2_
VII^g^	µ_3_-nic-κ^3^N:O:O'	6.680 (1)	CuN_3_O_2_
VIII^h^	µ_3_-nic-κ^3^N:O:O'	6.646 (2)	NiN_2_O_4_
IX^i^	µ_3_-nic-κ^3^N:O:O'	6.90 (1)	NiN_2_O_4_
X^j^	µ_3_-nic-κ^3^N:O:O'	6.622 (2)	NiN_2_O_4_
XI^k^	µ_3_-nic-κ^3^N:O:O'	7.324 (1)	MnN_2_O_4_
XII^l^	µ_3_-nic-κ^3^N:O:O'	6.890 (2)	NiN_2_O_4_
	µ_2_-nic-κ^2^N:O	7.027 (2)	NiN_3_O_3_

(a) [Cd(µ_2_-nic)(nic)(en)(H_2_O)]_2_.2H_2_O (this work); (b) [Cu(µ_2_-nic)(dien)]_2_(nic)_2_ (dien is diethylenetriamine) (Chen *et al.*, 2008); (c) [Cu(µ_2_-nic)(dien)]_2_(BF_4_)_2_.2MeOH [dien is diethylenetriamine] (Chen *et al.*, 2008); (d) [Mn_3_(µ_3_-nic)_4_(µ_2_-N_3_)_2_(H_2_O)_2_]_n_ (Chen *et al.*, 2001); (e) [Cd_3_(µ_3_-nic)_4_(µ_2_-N_3_)_2_(H_2_O)_2_]_n_ (Abu-Youssef, 2005); (f) [Cu_2_(µ_3_-nic)_2_(Me_2_bipy)_2_]_n_.2nClO_4_ [Me_2_bipy is 4,4'-dimethyl-2,2'-bipyridine] (Madalan *et al.*, 2005); (g) [Cu_2_(µ_3_-nic)_2_(bipy)_2_]_n_.2nClO_4_.2H_2_O [bipy is 2,2'-bipyridine] (Madalan *et al.*, 2005); (h) [Ni_4_(µ_3_-nic)_4_(µ_2_-nic)_4_(µ_2_-H_2_O)_2_]_n_.2nEtOH.2nH_2_O (Ayyappan *et al.*, 2001); (i) [Ni_4_(µ_3_-nic)_4_(µ_2_-nic)_4_(µ_2_-H_2_O)_2_]_n_ (Wu *et al.*, 2003); (j) [Ni_4_(µ_3_-nic)_4_(µ_2_-nic)_4_(µ_2_-H_2_O)_2_]_n_.2nH_2_O (Wasson & LaDuca, 2007); (k) [Mn(µ_3_-nic)_2_]_n_ (Lin *et al.*, 2000; Wang *et al.*, 2002); (l) [Ni_3_(µ_3_-nic)_2_(µ_2_-nic)_2_(µ_2_-N_3_)_2_(µ_2_-Hnic)_2_]_n_ (Liu *et al.*, 2005).
